# An Evaluation of Cation–Chloride Cotransporters NKCC1 and KCC2 in Carbamazepine-Resistant Rats

**DOI:** 10.3390/ijms26104764

**Published:** 2025-05-16

**Authors:** Cecilia Zavala-Tecuapetla, Sandra Orozco-Suárez, Angélica Vega-García, Joaquín Manjarrez-Marmolejo

**Affiliations:** 1 Experimental Laboratory of Neurodegenerative Diseases, National Institute of Neurology and Neurosurgery “Manuel Velasco Suárez”, Insurgentes Sur 3877, La Fama, Mexico City 14269, Mexico; manjarrezmj@yahoo.com.mx; 2 Medical Research Unit on Neurological Diseases, Specialty Hospital “Dr. Bernardo Sepúlveda”, National Medical Center “XXI Century”, Mexican Social Security Institute, Av. Cuauhtémoc 330, Doctores, Mexico City 06720, Mexico; sorozco5@hotmail.com (S.O.-S.); ange_li_k@hotmail.com (A.V.-G.)

**Keywords:** drug-resistant epilepsy, carbamazepine-resistance, cation–chloride cotransporter, NKCC1, KCC2, bumetanide, probenecid, adenosine receptor activation, NECA

## Abstract

Approximately one-third of epileptic patients do not respond adequately to drug therapy, leading to the development of drug-resistant epilepsy. Given the established role of dysregulated expression of two cation–chloride cotransporter proteins, NKCC1 and KCC2, in susceptibility to convulsion generation and epilepsy development, the present study evaluates the anticonvulsant potential of bumetanide (BUM, 10 mg/kg, i.p.) and probenecid (PROB, 50 mg/kg, i.p.), the potential of adenosine receptor activation (NECA, 1 mg/kg, i.p.) to modify the anticonvulsant efficacy of BUM, and the changes in NKCC1 and KCC2 protein expression levels in carbamazepine (CBZ)-resistant animals. In the window–pentylenetetrazole (PTZ) kindling model, male Wistar rats that undergo full kindling develop CBZ-resistance. The combination of BUM + PROB appears to have an anticonvulsant effect on CBZ-resistant convulsions, while alterations in the protein levels of the NKCC1 and KCC2 cotransporters are observed in CBZ-resistant animals. Despite the absence of therapeutic efficacy in managing convulsions through adenosine receptor activation (BUM + NECA), the activation of adenosine receptors exhibits the capacity to modulate the levels of the NKCC1 protein in the hippocampus of CBZ-resistant animals. This effect provides the initial evidence for a new therapeutic role of adenosine receptors in regulating the pathological levels of NKCC1 in drug-resistant epilepsy.

## 1. Introduction

Epilepsy, a neurological disease, is characterized by recurrent unprovoked seizures [1]. However, approximately one-third of individuals with epilepsy do not become seizure-free, despite the appropriate use of antiseizure medications (ASMs), and develop drug-resistant epilepsy [2,3].

In the search for improved therapeutic strategies, cation–chloride cotransporters have emerged as a focal point in research endeavors targeting epilepsy treatment [4,5].

Within this framework, neuronal chloride homeostasis is controlled by two major cation–chloride cotransporters: sodium–potassium–chloride cotransporter 1 (NKCC1) and potassium–chloride cotransporter 2 (KCC2). On the one hand, NKCC1 pumps Cl- into the cell, while KCC2 pumps Cl- out. Both functions are required for efficient synaptic inhibition [4,5].

Additionally, in the adult brain, dysregulated expression of NKCC1/KCC2 proteins appears to impact chloride homeostasis, neuronal excitability, and, consequently, the susceptibility to seizure generation [6,7]. While KCC2 shows a decreased expression, NKCC1 appears to increase in epilepsy [8,9]. Based on these findings, blocking NKCC1 with bumetanide (an FDA-approved diuretic drug) has been intensively evaluated as an anticonvulsant strategy in preclinical research [10]. However, bumetanide has demonstrated low brain penetration in adult animals, which could potentially impact its anticonvulsant efficacy in different animal models [11]. Previous evidence suggests that probenecid, a non-specific inhibitor of several transporters expressed at the blood–brain barrier (BBB), may increase the brain levels of bumetanide by inhibiting its active transport out of the brain [12]. The above could enhance the anticonvulsant effect of bumetanide.

On the other hand, a number of studies suggest that the permeability of the BBB can be modulated by adenosine signaling through the activation of adenosine receptors (A1R/A2AR) [13,14,15,16], allowing drugs to enter the brain. The aforementioned strategy could be considered a potential approach to enhance the brain penetration of bumetanide, thereby facilitating its potential anticonvulsant effects and facilitating treatment for drug-resistant epilepsy.

Therefore, the present study focused on evaluating the effects of bumetanide administration in carbamazepine (CBZ)-resistant animals, and on how the co-administration of probenecid or the activation of adenosine receptors (by NECA, a non-selective A1/A2A-R agonist) might improve the control of convulsions by bumetanide in CBZ-resistant animals. Finally, we assessed brain changes in NKCC1 and KCC2 expression in CBZ-resistant animals and how the activation of adenosine receptors could modify these cation–chloride cotransporters’ expression levels.

## 2. Results

### 2.1. Generation of CBZ-Resistant Animals

**Figure 1:**
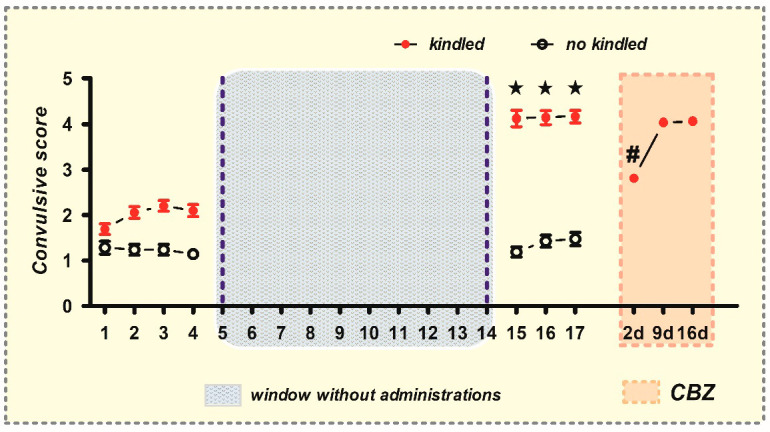
The development of chemical window kindling model and the subsequent generation of resistance to carbamazepine. The convulsive score was determined in rats following each administration of pentylenetetrazol (PTZ, 35 mg/kg, i.p.). The data include both non-kindled (black circles) and fully kindled (red points) animals (mean ± SEM). The administration of carbamazepine (CBZ; orange box) is indicated at 2, 9, and 16 days following the establishment of the fully kindled state. ★ *p* < 0.05, statistically significant difference from the initial assay in the kindled group. ^#^ *p* < 0.05, statistically significant difference from the kindled condition.

In the window–pentylenetetrazole (PTZ) kindling model, rats exhibited alterations in their convulsive behavior following the administration of PTZ (e.g., ear/facial twitching, sniffing, blinking, nodding, myoclonic body jerks, etc.). The behavioral score increased gradually over time, progressing from stage 1–3 to stage 4–5 convulsions (Figure 1). During the development of chemical kindling, 27% of the rats died due to generalized tonic–clonic convulsions. Conversely, no-kindled rats (19%) solely exhibited stage 1–2 convulsions during the last three PTZ injections, yielding a convulsive score of 1.5 ± 0.15 (Figure 1). Notably, the remaining rats (54%) achieved a fully kindled state (stage 4/5 convulsions), exhibiting a convulsive score of 4.2 ± 0.13 (Figure 1).

Subsequently, with these fully kindled animals (n = 61), we generated CBZ-resistant rats by administering a single dose of CBZ (40 mg/kg) at 2, 9, and 16 days after their last kindling stimulation (Figure 1). During the initial administration (2 days), CBZ effectively protected the kindled rats from stage 4/5 convulsions (convulsive score from 4.2 ± 0.13 to 2.8 ± 0.10 (*p* < 0.0001)) (Figure 1). However, subsequent administrations of CBZ on days 9 (second administration) and 16 (third administration) failed to elicit a significant impact on the convulsive score. Instead, the rats exhibited stage 4/5 convulsions, with a recorded convulsive score of 4.1 ± 0.10, thereby indicating the development of resistance to CBZ in these animals (Figure 1).

### 2.2. Effects of Bumetanide, Probenecid, and NECA on PTZ Induced Convulsions in CBZ-Resistant Rats

The objective of this study was to determine the efficacy of bumetanide, administered alone or in conjunction with probenecid or NECA, in suppressing PTZ-induced convulsions in CBZ-resistant rats.

**Figure 2:**
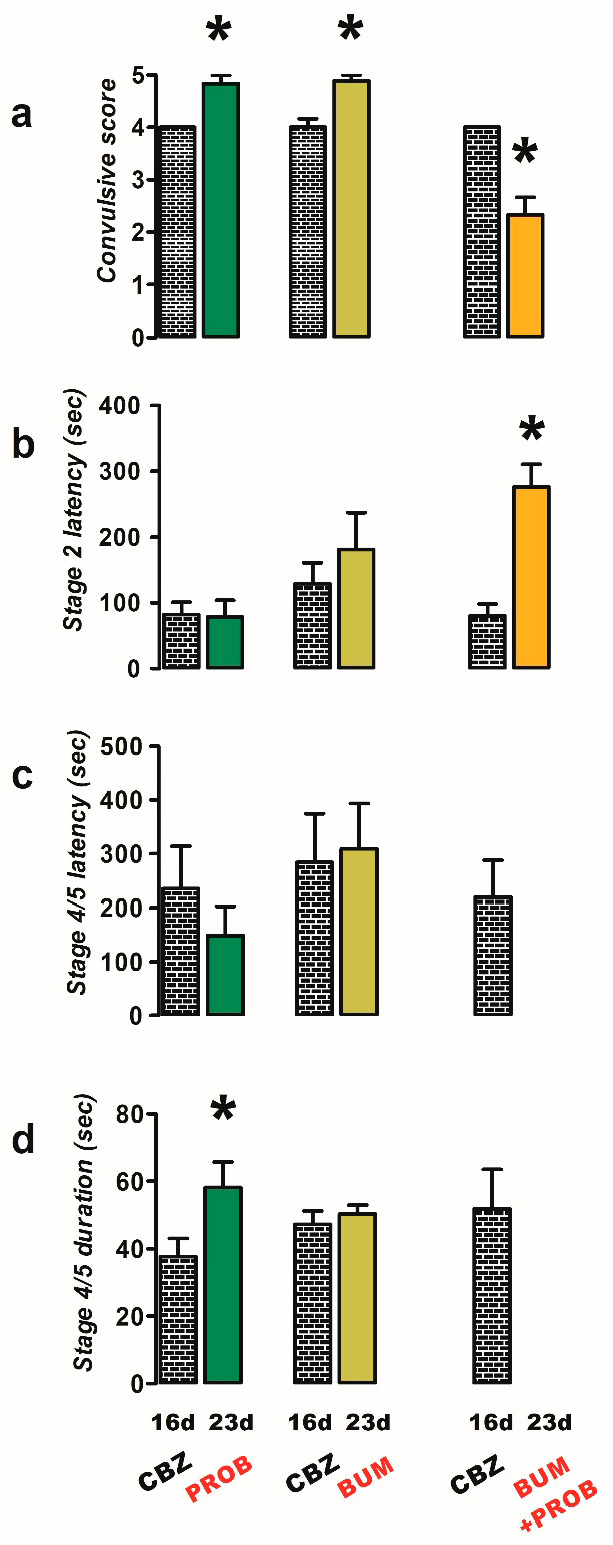
Effects of treatments with probenecid (PROB, 50 mg/kg i.p.; n = 6), bumetanide (BUM, 10 mg/kg i.p.; n = 9), and a combination of BUM and PROB (n = 6) against PTZ-induced convulsions in carbamazepine (CBZ)-resistant rats (23 day). The behavioral parameters evaluated included convulsive score (**a**), stage 2 latency (**b**), and the latency (**c**) and duration (**d**) of stage 4/5 convulsions. Data are presented as mean ± SEM. A statistically significant difference from the CBZ-resistant state (16 d) is indicated with an asterisk (* *p* < 0.05).

Initially, CBZ-resistant rats treated acutely with probenecid (PROB, 50 mg/kg; n = 6) predominantly exhibited stage 5 convulsions in response to PTZ stimulation (Figure 2a), resulting in an increased convulsive score (23 d, 4.8 ± 0.17) compared to the CBZ-resistant condition (16 d, 4.0 ± 0.0, *p* = 0.004; Figure 2a) and duration of stage 4/5 convulsions (23 d, 58.17 ± 7.6 s; 16 d, 37.67 ± 5.41 s; *p* = 0.013; Figure 2d). Probenecid did not modify significantly any other parameter evaluated (latency of convulsions; Figure 2b,c).

In rats resistant to CBZ, the administration of bumetanide (BUM, 10 mg/kg; n = 9) resulted in an elevated convulsive score (23 d, 4.9 ± 0.11) in comparison to the CBZ-resistant condition (16 d, 4.0 ± 0.17, *p* = 0.002; Figure 2a), without any observed impact on the other parameters.

However, the co-administration of bumetanide and probenecid (BUM + PROB, n = 6) led to a reduction in the severity of convulsions in response to PTZ stimulation (Figure 2a), as evidenced by the convulsive score (23 d, 2.3 ± 0.33) compared to the CBZ-resistant condition (16 d, 4.0 ± 0.0, *p* = 0.004; Figure 2a), preventing the generalization of convulsions (Figure 2c,d) and increasing the latency to stage 2 convulsions (23 d, 276.0 ± 34.84 s; 16 d, 80.33 ± 17.9 s; *p* = 0.017; Figure 2b).

Next, we investigated whether probenecid, bumetanide, or their co-administration could potentiate CBZ’s anticonvulsant effect in CBZ-resistant rats.

**Figure 3:**
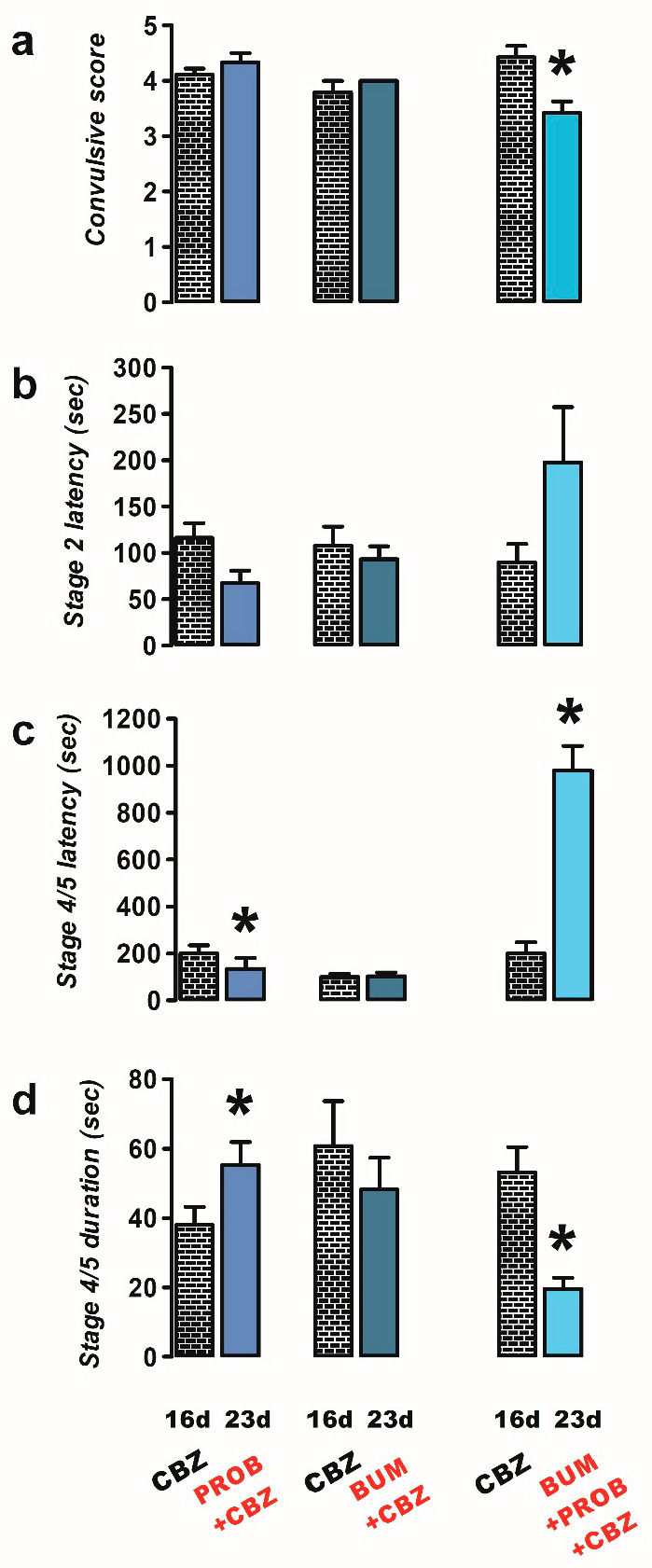
Effects of treatments of probenecid (PROB, 50 mg/kg i.p.; n = 9), bumetanide (BUM, 10 mg/kg i.p.; n = 6), and a combination of BUM + PROB (n = 7) with carbamazepine (CBZ, 40 mg/kg i.p.) against PTZ-induced convulsions in CBZ-resistant rats (23 day). The behavioral parameters evaluated included convulsive score (**a**), stage 2 latency (**b**), and the latency (**c**) and duration (**d**) of stage 4/5 convulsions. Data are presented as mean ± SEM. A statistically significant difference from the CBZ-resistant state (16 d) is indicated with an asterisk (* *p* < 0.05).

The administration of PROB combined with CBZ (n = 9) exhibited a proconvulsant effect similar to that observed with PROB alone. Specifically, there was a decrease in latency (23 d, 134.2 ± 47.7 s) and an increase in duration (23 d, 55.3 ± 6.54 s) of stage 4/5 convulsions in the PROB + CBZ group compared to the CBZ-resistant condition (latency, 16 d, 200.4 ± 35.10 s, *p* = 0.032; duration, 16 d, 38.0 ± 5.15 s, *p* = 0.012; Figure 3c,d).

Neither the latency nor the duration of stage 2 or 4/5 convulsions was modified with the administration of BUM + CBZ (n = 6) compared to the CBZ-resistant condition (Figure 3a–d).

However, when PROB + BUM (n = 7) was co-administered with CBZ, a decrease in the convulsive score was observed (23 d, 3.4 ± 0.20, *p* = 0.018; Figure 3a). A significant increase in the latency of the convulsions was only observed for stage 4/5 convulsions (23 d, 980.7 ± 103.7 s, *p* = 0.0001; Figure 3b,c), and there was a significant decrease in the duration of stage 4/5 convulsions (23 d, 19.7 ± 3.18 s, *p* = 0.014; Figure 3d) compared to the CBZ-resistant condition (convulsive score, 16 d, 4.43 ± 0.20; stage 4/5 latency, 16 d, 200.4 ± 47.7 s; stage 4/5 duration, 16 d, 53.14 ± 7.30 s; Figure 3a–d).

**Figure 4:**
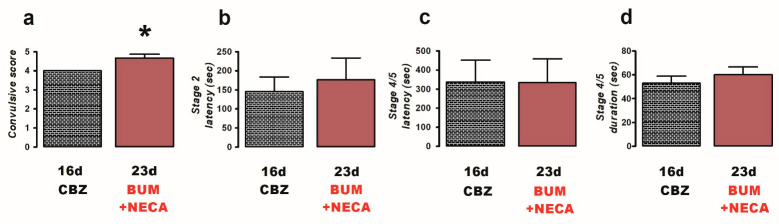
Effects of treatment with bumetanide (BUM, 10 mg/kg i.p.) and NECA (1 mg/kg i.p., 8 h) (n = 6) against PTZ-induced convulsions in carbamazepine (CBZ)-resistant rats (23 day). The behavioral parameters evaluated included convulsive score (**a**), stage 2 latency (**b**), and the latency (**c**) and duration (**d**) of stage 4/5 convulsions. Data are presented as mean ± SEM. A statistically significant difference from the CBZ-resistant state (16 d) is indicated with an asterisk (* *p* < 0.05).

Finally, the co-administration of BUM (10 mg/kg) + NECA (1 mg/kg) (n = 6) was evaluated to investigate the potential of adenosine receptor activation to enhance the anticonvulsant efficacy of BUM in CBZ-resistant rats. The result of the co-administration of BUM + NECA was a worsening of convulsions, as indicated by an increase in the convulsive score (23 d, 4.7 ± 0.21, *p* = 0.025; Figure 4a) compared to the CBZ-resistant condition (16 d, 4.0 ± 0.0; Figure 4a). No other parameters demonstrated significant alterations (Figure 4b–d).

### 2.3. Changes in Protein Expression Levels of NKCC1 and KCC2 in Rat Brain Regions

A Western blot analysis was employed to assess whether the protein expression levels of these two cation–chloride cotransporters (NKCC1 and KCC2) were modified or not under different experimental conditions.

#### 2.3.1. NKCC1 in the Hippocampus

**Figure 5:**
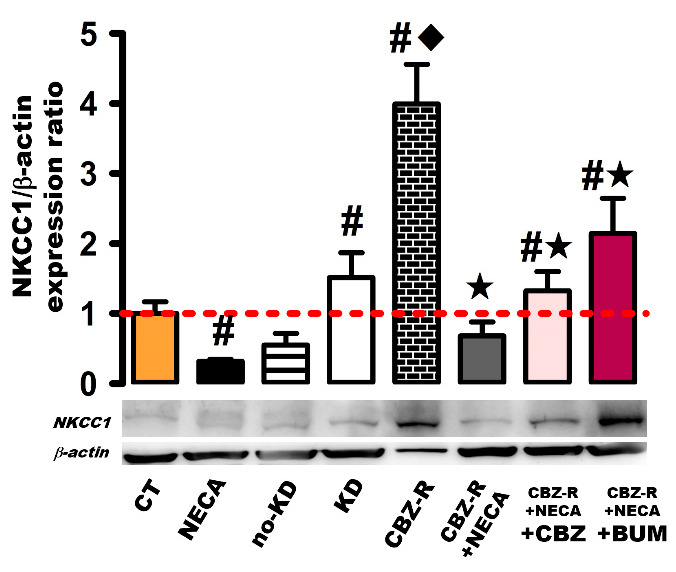
Expression of the NKCC1 protein in the hippocampus of the rat. β-actin was used as loading control. The protein expression levels were calculated as ratios of NKCC1/β-actin and normalized to the values obtained in the control group. The red dashed line indicates basal expression levels. The data represent the means ± SEM of three animal samples. ^#^ *p* < 0.05 indicates a statistically significant difference from the control (CT) condition. ^◆^ *p* < 0.05 indicates a statistically significant difference from the kindled (KD) group. ★ *p* < 0.05 indicates a statistically significant difference from the CBZ-resistant (CBZ-R) group. CBZ: carbamazepine; BUM: bumetanide.

Basal NKCC1 protein levels were significantly decreased with NECA (8 h post-treatment; 70%; 0.30 ± 0.01, *p* < 0.001) (Figure 5). In contrast, the kindled and CBZ-resistant groups exhibited augmented NKCC1 protein levels (50%, 1.52 ± 0.36, *p* < 0.001; 300%, 4.0 ± 0.57, *p* < 0.003, respectively) compared to the control group (Figure 5). A more pronounced increase was observed in the CBZ-resistant group compared to the kindled group (*p* < 0.003) (Figure 5). However, the administration of NECA (8 h post-treatment) to the CBZ-resistant animals resulted in a significant decrease in NKCC1 protein levels, returning them to their basal levels (0.68 ± 0.20, *p* < 0.003) (Figure 5).

The administration of CBZ to animals previously treated with NECA and resistant to CBZ resulted in an increase in NKCC1 protein levels (33%, 1.33 ± 0.28, *p* < 0.003) compared to the control group (Figure 5). However, this increase was not as significant as the one observed in the CBZ-resistant group. A comparable effect was seen with the administration of BUM, where the NKCC1 protein levels increased (114%, 2.14 ± 0.51, *p* < 0.003) compared to the control group (Figure 5), though this increase was less pronounced than that observed in the CBZ-resistant group. No significant changes were detected in the no-kindled group (Figure 5).

#### 2.3.2. KCC2 in the Hippocampus

**Figure 6:**
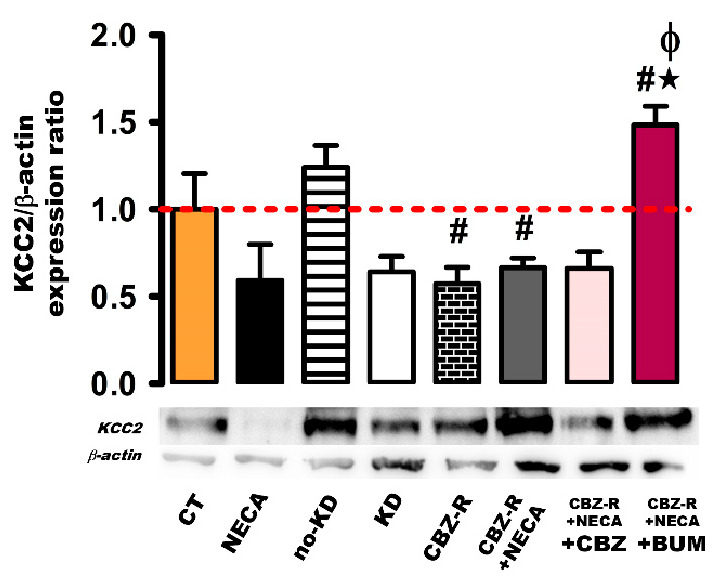
Expression of the KCC2 protein in the hippocampus of the rat. β-actin was used as loading control. The protein expression levels were calculated as ratios of KCC2/β-actin and normalized to the values obtained in the control group. The red dashed line indicates basal expression levels. The data represent the means ± SEM of three animal samples. ^#^ *p* < 0.05 indicates a statistically significant difference from the control (CT) condition. ★ *p* < 0.05 indicates a statistically significant difference from the CBZ-resistant (CBZ-R) group. Φ *p* < 0.05 indicates a statistically significant difference from the CBZ-R group treated with NECA. CBZ: carbamazepine; BUM: bumetanide.

The effects of NECA on basal KCC2 protein levels were not significant (8 h post-treatment), nor were they modified in the no-kindled group compared to the control group (Figure 6). In the kindled group, the KCC2 protein levels seemed to decrease, but this change did not reach statistical significance (Figure 6). However, a significant decrease in KCC2 protein levels was observed in both the CBZ-resistant group and the CBZ-resistant group treated with NECA (8 h), compared to the control group (43%, 0.57 ± 0.10, *p* < 0.004 and 34%, 0.67 ± 0.05, *p* < 0.003) (Figure 6). This observed decrease was consistent across both groups. Regarding the administration of BUM to the CBZ-resistant group treated with NECA, BUM increased the KCC2 protein levels (48%, 1.48 ± 0.11, *p* < 0.004) compared to the control group. Additionally, BUM increased the KCC2 protein levels (91% and 81%, *p* < 0.004) compared to the CBZ-resistant group and the CBZ-resistant group treated with NECA (Figure 6).

No statistically significant changes were observed in the NECA-treated CBZ-resistant animals administered with CBZ (Figure 6).

#### 2.3.3. KCC2 in the Cortex

**Figure 7:**
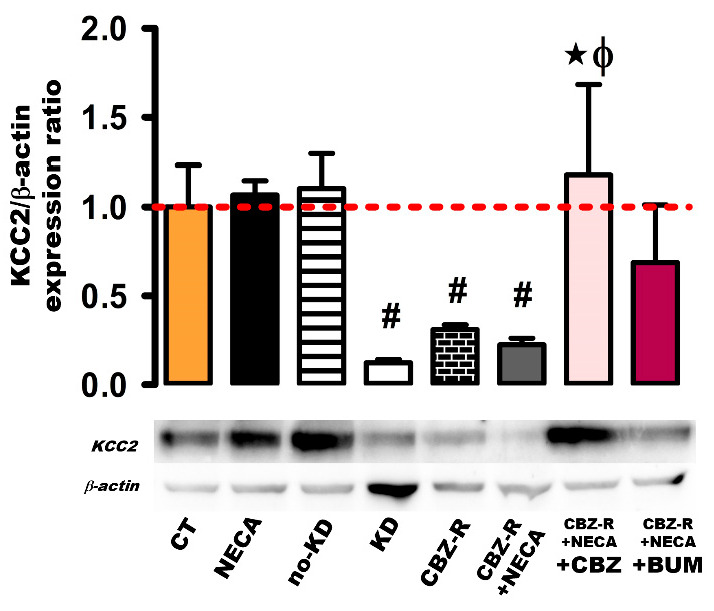
Expression of the KCC2 protein in the cortex of the rat. β-actin was used as loading control. The protein expression levels were calculated as ratios of KCC2/β-actin and normalized to the values obtained in the control group. The red dashed line indicates basal expression levels. The data represent the means ± SEM of three animal samples. ^#^ *p* < 0.05 indicates a statistically significant difference from the control (CT) condition. ★ *p* < 0.05 indicates a statistically significant difference from the CBZ-resistant (CBZ-R) group. Φ *p* < 0.05 indicates a statistically significant difference from the CBZ-R group treated with NECA. CBZ: carbamazepine; BUM: bumetanide.

With respect to the cortex region, the basal KCC2 protein levels remained unchanged in both the NECA (8 h post-treatment) and no-kindled groups (Figure 7). However, a significant decrease in the KCC2 protein levels was observed in the kindled group, the CBZ-resistant group, and the NECA-treated CBZ-resistant animals (87%, 0.13 ± 0.018, *p* < 0.012; 69%, 0.31 ± 0.028, *p* < 0.013, and 77%, 0.23 ± 0.04, *p* < 0.012, respectively) compared to the control group (Figure 7). In the CBZ-resistant animals treated with NECA and subsequently administered with CBZ, the KCC2 protein levels increased to the basal levels (1.18 ± 0.50, *p* < 0.012) (Figure 7). However, when the CBZ-resistant group treated with NECA was administered with BUM, there was no significant modification of the KCC2 protein levels (0.69 ± 0.33) (Figure 7).

## 3. Discussion

In this study, we demonstrated that the concomitant administration of bumetanide (BUM) and probenecid (PROB) demonstrated anticonvulsant properties in CBZ-resistant animals. This was evidenced by the prevention of convulsion generalization and a significant delay in the onset of focal convulsions. Furthermore, our findings revealed alterations in the protein expression levels of NKCC1 and KCC2 cotransporters in CBZ-resistant animals, thereby substantiating the involvement of these cation–chloride cotransporters in CBZ resistance. In addition to the aforementioned points, the administration of NECA (non-selective A1/A2A-R agonist) demonstrated a direct modulation of hippocampal NKCC1 protein levels in CBZ-resistant animals by activating the adenosine receptors. This effect suggests a new therapeutic role for adenosine receptors in controlling pathological levels of NKCC1 in drug-resistant epilepsy, which has not been previously demonstrated.

First, BUM and PROB have been demonstrated to induce proconvulsant effects, as evidenced by the augmentation of both severity and duration of convulsions in CBZ-resistant animals. Although the dose of PROB (50 mg/kg i.p.; 50 min prior to stimulation with PTZ) that was utilized in this study has been reported to have no effect on kindled rats [17,18], a proconvulsant effect was observed in CBZ-resistant animals when PROB was administered alone. These discrepancies in the reported outcomes could be attributed to the potential contributions of different factors. First, it is important to note that the drug-resistance condition encompasses pathophysiological mechanisms other than those involved only in the development of the kindled state. These mechanisms may contribute to the proconvulsant effect observed with PROB. Furthermore, PROB appears to exhibit additional effects on other molecular targets [19,20], which could also account for its proconvulsant effects observed in CBZ-resistant animals. Finally, the administration time (30 or 75 min vs. 50 min) [17,18], the dosage administered (high doses vs. 50 mg/kg) [20], and the administration scheme (acute vs. single) [20] may also be factors that influence the observed effects with PROB. Further investigation is necessary to address these issues.

Then, with regard to BUM (10 mg/kg, i.p.; NKCC1/NKCC2 antagonist), given that NKCC1 is expressed in both neurons and astrocytes [21], the pharmacological effect of s systemic administration of BUM could result from the inhibition of NKCC1 in both cell types (neurons/astrocytes), where it has been demonstrated that astrocytic NKCC1 has an anticonvulsant role, while neuronal NKCC1 has a proconvulsant role [6]. Due to these dual effects of BUM, it is possible to explain the lack of therapeutic efficacy in controlling convulsions in our CBZ resistance model. Furthermore, the poor brain penetration of BUM (a loop diuretic) [22,23] suggests that its proconvulsant effect may be attributable to its actions on NKCC1 outside the brain. However, the underlying mechanism of the proconvulsant effect of BUM after its single systemic administration (i.p.) remains to be elucidated [24], as BUM appears to exhibit additional effects on other molecular targets [11].

On the other hand, as reported in the literature, PROB has been observed to potentiate phenytoin’s anticonvulsant effect in fully kindled rats [17,18]. In the present study, a proconvulsant effect was observed with the coadministration of PROB + CBZ in CBZ-resistant animals, promoting generalization of convulsions and increasing their duration. This result is similar to that observed with the administration of PROB alone, suggesting that PROB itself does not have any potentiating effect on the administration of CBZ. The co-administration of BUM + CBZ in CBZ-resistant animals had no effect.

In the meantime, the co-administration of both compounds (BUM + PROB) exhibited an anticonvulsant effect, as characterized by the prevention of generalization of convulsions, as well as the delay and reduction in severity of focal convulsions, in CBZ-resistant animals. This anticonvulsant effect may be attributable to the inhibition of BUM’s active transport from the brain by PROB [12]. Given the poor brain penetration of BUM [22,23], several strategies have been investigated to enhance its brain levels [12,23,25,26]. One such strategy involves the administration of PROB, which has been documented as a non-selective inhibitor of various transporters (e.g., organic anion transporters, multidrug resistance proteins, monocarboxylate transporters, among others) [17,18,23,27,28], thereby increasing the brain levels of BUM [12,23,29]. This could allow BUM to reach the appropriate brain levels to have an effect on the NKCC1 cotransporter and, consequently, generate an anticonvulsant effect in CBZ-resistant animals. In order to corroborate the aforementioned points, it is necessary to evaluate the brain levels of BUM, as well as the type of transporter that could be associated with this action in CBZ-resistant animals, in subsequent studies.

A further point that merits consideration is that a previous report indicated alterations in the expression of the BBB’s transporters, including P-glycoprotein (P-GP) and multidrug resistance proteins (MRPs), in CBZ-resistant rats [15]. It is plausible that pathophysiological or compensatory changes in the expression of other BBB transporters associated with CBZ resistance influence the effect of PROB in these animals.

Subsequently, an anticonvulsant effect was observed in CBZ-resistant animals following the administration of BUM + PROB with CBZ. This effect was characterized by a delay in the onset of generalized convulsions and a reduction in their duration. However, in these animals, the generalization of convulsions was not prevented, as was observed in the BUM + PROB group. This finding suggests that the administration of CBZ in these animals disrupts the protective effect of the BUM + PROB combination, and that CBZ exerts its effect by promoting generalized convulsions. Consequently, the condition of resistance to CBZ persists in these animals.

Certainly, given the established role of adenosine signaling in modulating BBB permeability through the activation of adenosine receptors (A1R/A2AR) [13,14,15,16], the administration of NECA could potentially enhance the brain penetration of BUM, which could consequently improve the management of convulsions. However, in our study, the co-administration of BUM + NECA exhibited an effect comparable to that observed with BUM alone, a proconvulsant effect that exacerbates convulsion severity in CBZ-resistant animals. Our previous research demonstrated that the administration of NECA alone in CBZ-resistant rats resulted in a delay in the onset of convulsions [15], an effect that could be associated with the brain activation of adenosine receptors [30,31]. Consequently, it can be inferred that the proconvulsant effect observed with the co-administration of BUM + NECA is attributable solely to the action of BUM, and that NECA was ineffective in enhancing the control of convulsions by BUM in CBZ-resistant animals.

Up to this point, it is important to emphasize that the results concerning the drugs evaluated have been based on observed behavioral changes. We do not rule out a further analysis of the electroencephalogram (EEG) correlate of the behavioral changes in this model. Indeed, the EEG is a crucial tool in evaluating the effectiveness of potential drugs against drug-resistant seizures. Our future aim is to evaluate the EEG activity during the development of the window–PTZ kindling model, the generation of CBZ-resistance, and after drug treatment, to understand the epileptic processes at each stage.

Additionally, with regard to the protein expression levels of the cation–chloride cotransporters NKCC1 and KCC2, we observed comparable alterations to those previously documented in preclinical and clinical studies.

For instance, increased protein levels of NKCC1 in the hippocampus from fully kindled and CBZ-resistant animals have been observed, which coincide with previous results from epileptic patients [9,32,33]. This finding is further supported by the results of animal models [34,35,36,37,38]. It is noteworthy that the increase in NKCC1 protein levels was significantly more pronounced in CBZ-resistant animals, underscoring the pivotal role of this cotransporter in the development of drug resistance.

With respect to the KCC2 protein levels, a similar decrease was observed between fully kindled and CBZ-resistant animals in both the hippocampus and the cortex. These results are consistent with those previously reported in human epileptic tissue and epilepsy animal models [8,32,34,35,36,38,39,40,41,42,43].

However, the most significant finding emerged regarding NECA (a nonselective A1/A2A-R agonist), which modulated the NKCC1 protein levels in the hippocampus after 8 h, both under physiological conditions (as observed in control animals administered with NECA) and under pathophysiological conditions, such as CBZ resistance where the restoration of basal levels was observed. This could represent a new therapeutic strategy to regulate excitability in this brain area and treat drug-resistant epilepsy. For this reason, further investigation is necessary to understand how adenosine receptor activation affects NKCC1 protein levels and if these receptors actually mediate the effect. An adenosine receptor antagonist (A1/A2A-R) should be administered to CBZ-resistant animals to evaluate if it prevents the NECA’s effects on NKCC1 levels in the hippocampus. The next step is to identify the receptor subtype responsible for regulating NKCC1 levels, which will be achieved by administering specific agonists of the adenosine A1 and A2A receptor subtypes.

As has been reported, the activation of adenosine receptors can reduce the protein levels of P-GP (an efflux transporter) [14,15] through different molecular mechanisms (e.g., ubiquitination and translocation) in a time-dependent and reversible manner [14]. Therefore, it is plausible that similar mechanisms [44] may be involved in the reduction of NKCC1 protein levels through adenosine receptor activation. However, further investigation is necessary to elucidate the precise mechanisms and the nature of the reversible and time-dependent effect.

On the other hand, the co-administration of CBZ or BUM with NECA demonstrated effects on the expression of both cotransporters (NKCC1 and KCC2) in CBZ-resistant animals in both the hippocampus and the cortex.

In the presence of NECA, CBZ was found to increase NKCC1 protein levels once more. Furthermore, CBZ was able to restore protein basal levels of KCC2 in the cortex, potentially as a compensatory response to counteract the resistant condition in CBZ-resistant rats. The observed changes in NKCC1/KCC2 protein levels may be associated with the behavioral modulation induced by NECA + CBZ, as previously reported [15]. Specifically, NECA + CBZ delayed and controlled convulsion spreading in CBZ-resistant rats. That is, there was restoration of the CBZ’s anticonvulsant effect [15]. The mechanisms that regulate the expression of the cotransporters mediated by adenosine receptor (A1/A2A-R) activation and CBZ remain to be established.

In the context of BUM, it has been reported that treatment with BUM can affect the protein expression levels of the NKCC1 cotransporter. Specifically, it has been observed to block the pathophysiological upregulation of NKCC1, thereby contributing to the anticonvulsant efficiency of BUM [33,36]. However, the present study found that increased NKCC1 expression after treatment with BUM is promoted in the presence of adenosine receptor activation by NECA in resistance conditions, which is associated with the proconvulsant effect of BUM observed in CBZ-resistant rats treated with BUM + NECA.

Furthermore, we found that BUM also increased hippocampal KCC2 protein expression levels above the basal levels in the presence of NECA in resistance conditions. Given that NECA does not appear to affect KCC2 expression under physiological conditions, it can be inferred that the observed increase in KCC2 expression is a result of the effects of BUM. Previous studies have shown that BUM alone can restore the basal levels of KCC2 in the rat pilocarpine model [36], which is consistent with the findings of this study, where the KCC2 levels were found to be above baseline under drug resistance conditions. A more detailed investigation is necessary to determine the mechanisms by which BUM promotes the upregulation of NKCC1 or KCC2 cotransporters in CBZ-resistant animals. A primary question to be answered is whether BUM altered the basal expression levels of NKCC1/KCC2 in healthy animals.

However, it is important to note that there are some side effects that limit the use of BUM in chronic treatments, such as in the case of drug-resistant epilepsy [24]. Beyond its poor brain penetration, BUM causes diuresis, hypokalemic alkalosis, and hearing loss [24,45,46]. Consequently, there is an ongoing need to develop alternative pharmaceutical agents that do not exhibit these adverse effects.

Finally, the KCC2 cotransporter has recently been implicated as a possible contributing factor to the development of resistant seizures to some antiseizure drugs (e.g., resistance to benzodiazepines or valproate) [47,48,49]. Furthermore, it has been suggested that the KCC2 cotransporter may contribute to the increased risk of SUDEP (sudden unexpected death) in drug-resistant epileptic patients [7]. Consequently, strategies aimed at counteracting the observed decrease in KCC2 levels in our drug resistance model, through the implementation of pharmacological or genetic interventions [47,48,49], hold considerable potential for the restoration of neuronal excitability homeostasis in drug-resistant epilepsy.

## 4. Materials and Methods

**Figure 8:**
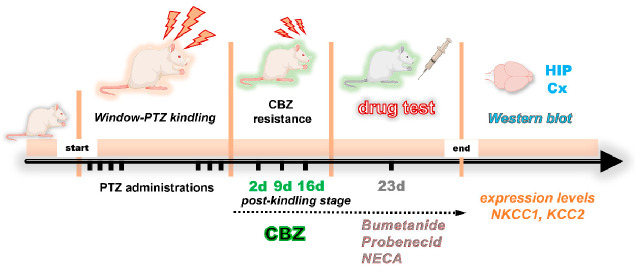
The following timeline delineates the developmental process of the window–PTZ kindling model, including the generation of resistance to carbamazepine (CBZ) during the postkindling stage, pharmacological testing of different drugs, and evaluation of protein levels of the cation–chloride cotransporters (NKCC1/KCC2).

As illustrated in the experimental timeline (Figure 8), the methodology employed in the development of this study is summarized.

### 4.1. Animals

A total of 118 male Wistar rats, with a weight range of 250 to 300 g, were utilized in this study. The animals were provided and housed at the National Institute of Neurology and Neurosurgery’s animal house (INNN-MVS, Mexico City, Mexico), where they were maintained in a 12:12 h light/dark cycle at a constant temperature (22 ± 1 °C) and humidity (60 ± 10%). The animals had free access to water and a standard pellet diet. The body weights and well-being of the animals were monitored on a three-times-per-week basis. To avoid any potential confusion, each experimental group was housed separately in distinct cages with standard laboratory bedding. Each cage was marked with a unique sign and accompanied by straightforward instructions detailing the treatment under evaluation. The sample size was predetermined based on the model’s extant experience and in accordance with the principles of the Three Rs [15,50,51]. All experimental protocols were carried out in accordance with the Internal Committee for the Care and Use of Laboratory Animals (CICUAL-INNN; project 35/23) and in accordance with the Mexican Official Norm (NOM-062-ZOO-1999). Every effort was made to minimize both the suffering and the number of animals used [51].

### 4.2. Drugs and Chemicals

Pentylenetetrazole (PTZ, GABAA receptor antagonist) was freshly dissolved in a 0.9% physiological saline solution. Carbamazepine (CBZ, sodium channel blocker) and 5′-(N-ethylcarboxamido) adenosine (NECA, non-selective A1/A2A-R agonist) were dissolved in 0.3% dimethyl sulfoxide (DMSO, *v*/*v*). Bumetanide (NKCC1/NKCC2 antagonist) and probenecid (unspecific transport inhibitor) were dissolved in saline by means of dilute NaOH [12]. All drugs were purchased from Sigma (Sigma-Aldrich, St. Louis, MO, USA).

### 4.3. Development of Chemical Kindling Model and the Induction of Drug Resistance

As previously reported [15,51], the window–PTZ kindling model has characteristics that make it technically simple and minimally invasive in rodents, which facilitates the generation of drug-resistant animals (a significant number of drug-resistant rats are produced). We have used this animal model as an alternative preclinical approach to predict the effectiveness of potential drugs against drug-resistant seizures [15,50].

Prior to the initiation of the model, the animals were habituated to daily handling and the laboratory environment, with the objective of avoiding any discomfort. The window–PTZ kindling model was induced by the administration of subconvulsive doses of PTZ (35 mg/kg i.p.; 1 mL/kg body weight) three times per week, following the previously reported PTZ administration scheme of 17 assays (four initial administrations followed by 10 assays without PTZ administrations, and ending with three administrations of PTZ) [15,52]. Convulsive behavior was monitored for 20 min following each administration of PTZ, according to the modified Racine scale [53,54], as follows: Stage 0: no response; Stage 1: ear and facial twitching, sniffing, and blinking; Stage 2: nodding or myoclonic body jerks; Stage 3: unilateral forelimb clonus, bilateral forelimb clonus; Stage 4: rearing with bilateral forelimb clonus; Stage 5: generalized tonic–clonic convulsions with loss of postural control. Animals that exhibited three consecutive Stage 4 or 5 convulsions following the administration of the final PTZ injections were designated as fully kindled rats, while rats that did not present them were designated as no-kindled [53,54].

It is noteworthy that, during the development of the model, the only recorded phenomenon was the death of some animals that presented generalized tonic–clonic seizures. The animals exhibited no discernible alterations in behavior (e.g., decreased activity, acute vocalizations, weight loss, diarrhea, among others) that would necessitate the implementation of humane endpoints.

Subsequently, the rats that had been kindled were administered a single dose of CBZ (40 mg/kg, intraperitoneal (i.p.), 60 min prior to PTZ) on days 2, 9, and 16 following their last kindling stimulation. This procedure has been described in detail in previous reports (see [15,55]). The convulsive behavioral evaluation included parameters such as convulsion severity (according to the Racine scale), latency (period of time) to the onset of Stage 2 (focal) or 4/5 (generalized) convulsions, and duration of convulsions (period of time) of Stage 4/5.

### 4.4. Pharmacological Evaluation Design

CBZ-resistant animals were divided into seven groups through the implementation of a simple randomization method to assess whether the tested drugs could have anticonvulsant effects on these animals:

1. Probenecid group (n = 6): one week after the last administration of CBZ (day 23), rats were administered probenecid (50 mg/kg i.p.) 50 min prior to stimulation with PTZ;
2. Bumetanide group (n = 9): these animals were treated as above, except that the rats were administered with bumetanide (10 mg/kg i.p.) 20 min prior to stimulation with PTZ;
3. Probenecid + bumetanide group (n = 6): rats were administered with both probenecid and bumetanide, as specified in the previous groups;
4. Probenecid + CBZ (n = 9): rats were administered probenecid (as specified in group 1) and CBZ (40 mg/kg i.p., 60 min before PTZ), respectively;
5. Bumetanide + CBZ (n = 6): rats were treated as previously described, with the exception that they were administered bumetanide (10 mg/kg i.p.) and CBZ (40 mg/kg i.p.), as specified in the preceding groups;
6. Bumetanide + probenecid + CBZ (n = 7): rats were treated with bumetanide (10 mg/kg i.p.), probenecid (50 mg/kg i.p.), and CBZ (40 mg/kg i.p.), as specified in the previous groups;
7. Bumetanide + NECA (n = 6): rats were administered with both bumetanide (10 mg/kg i.p.) and NECA (1 mg/kg i.p., 8 h prior to stimulation with PTZ).

The doses of probenecid, bumetanide, and NECA used in this study were determined based on prior research conducted in rats [15,18,31,56]. In these experiments, the evaluators were unaware of the treatments. The convulsive behavioral evaluation included parameters such as convulsion severity, latency to the onset of Stage 2 (focal) or 4/5 (generalized) convulsions, and the duration of convulsions of Stage 4/5.

### 4.5. Western Blot

A Western blot analysis was conducted on the brain tissue to assess the protein expression levels of NKCC1 and KCC2 in various animal groups (n = 3):

1. CT: control rats that received a saline solution only;
2. NECA: rats that received a single administration of NECA (1 mg/kg, i.p.) and were sacrificed 8 h later;
3. no-KD: no kindled animals, animals that did not present three consequent Stage 4 or 5 convulsions during the last three PTZ injections;
4. KD: fully kindled rats;
5. CBZ-R: carbamazepine resistant rats;
6. CBZ-R + NECA: CBZ-resistant rats treated with a single administration of NECA (1 mg/kg, i.p.) and sacrificed 8 h later;
7. CBZ-R + NECA + CBZ: CBZ-resistant rats treated with a single administration of NECA (1 mg/kg, i.p.) and CBZ (40 mg/kg, i.p.);
8. CBZ-R + NECA + BUM: CBZ-resistant rats treated with a single administration of NECA (1 mg/kg, i.p.) and bumetanide (10 mg/kg, i.p.).

The brain regions, including the cortex and hippocampus, were meticulously dissected and stored at −70 °C until processing. Initially, the protein extraction from the tissue was carried out using a lysis buffer composed of 50 mM Tris-HCl (pH 8.0), 150 mM sodium chloride, 1.0% IGEPAL CA-630 (NP-40), 0.5% sodium deoxycholate, and 0.1% sodium dodecyl sulfate (Sigma-Aldrich, St. Louis, MO, USA), plus with added protease inhibitors (cOmplete™, Mini, Protease Inhibitor Cocktail, EDTA free, 11836170001, Roche, Sigma-Aldrich, St. Louis, MO, USA) (Complete Mini, EDTA free, 11836170001, Roche). Each tissue sample was homogenized by using a 300 μL lysis buffer/300 mg tissue. Subsequently, the samples were subjected to centrifugation at 12,500 rpm for 40 min at 4 °C. The resultant pellet was then recovered, and the total protein content was measured using the Bradford protein assay (Bradford assay, Bio-Rad Laboratories Inc., Irvine, CA, USA).

The protein samples (60 μg) were separated by 6 and 8% SDS-PAGE gels (80 V, 2.75 h). Subsequently, the gel was transferred to nitrocellulose membranes (90 V, 3 h, room temperature) by a wet chamber (Mini Trans-Blot Central Core, Bio-Rad) using a transfer buffer (0.192 M glycine, 0.025 M Tris-Base, 20% methanol). The nitrocellulose membranes underwent a two-hour blocking step with 10% milk powder in PBS with Tween-20 (0.15 M PBS, 0.1% Tween-20) prior to a 48-h incubation with respective primary antibodies, such as rabbit anti-NKCC1 (1:1000; 130 kDa; ab59791, Abcam, Cambridge, UK) and rabbit anti-KCC2 (1:6000; 123 kDa; ab49917, Abcam, Cambridge, UK). The membranes were then washed (3 times, 5 min) and incubated for 2 h with the horseradish peroxidase-conjugated secondary antibody: goat anti-rabbit IgG antibody (H + L) peroxidase (HRP) (1:10,000; PI-1000-1, Vector Laboratories Inc., Newark, CA, USA). Proteins were detected by a chemiluminescence reaction (LuminataTM Crescendo Western HRP substrate, Millipore Corp, Burlington, MA, USA).

The mouse monoclonal β-Actin antibody (C4, sc-47778, Santa Cruz Biotechnology, Inc., Dallas, TX, USA) was diluted to a concentration of 1:1000 and utilized as a loading control. Finally, a densitometric analysis was performed using ImageJ 1.52i (NIH, Bethesda, MD, USA), ImageJ, with the optical density of each protein sample being calculated according to the reference bands of β-actin. The proteins were expressed as the ratios of cotransporters/β-actin (NKCC1/β-actin, KCC2/β-actin) and then normalized relative to the values measured in the control group.

### 4.6. Statistical Analysis

Statistical analysis was performed using GraphPad Prism 5.01 (GraphPad Software, La Jolla, CA, USA). The experimental data from each group were included in the analysis. All data were plotted as mean values, with error bars representing the standard error mean (S.E.M.). The significance level was set at *p* < 0.05. The Kolmogorov–Smirnov test was used for checking normality before performing the statistical tests. The kindling development results were analyzed using a one-way ANOVA with repeated measures and Tukey’s post hoc test for multiple comparisons. To assess the differences between the CBZ-resistant condition (16 d) and the drug treatment (23 d) for each drug, Student’s *t*-test for paired replicates was employed. The alterations in protein levels of cation–chloride cotransporters in the rat brain areas were analyzed using a one-way ANOVA followed by Tukey’s post hoc test for multiple comparisons.

## 5. Conclusions

The results of the present study indicate three key points. First, an observation was made of dysregulation of NKCC1/KCC2 expression, which may contribute to CBZ resistance. Second, the combination of bumetanide (BUM) and probenecid (PROB) demonstrated anticonvulsant efficacy in CBZ-resistant animals. Third and most notably, the study revealed that activation of adenosine receptors appears to modulate the expression of the NKCC1 cotransporter.

It is noteworthy that a number of studies have indicated that the modulation of NKCC1/KCC2 expression may serve as a therapeutic strategy for drug-resistant epilepsy [33,36,48,50]. This suggests that the activation of adenosine receptors could represent a novel avenue for the regulation of these expression levels. However, further research is necessary to ascertain its usefulness in this area.

## Data Availability

Data are available on request from the corresponding author.

## References

[1] Fisher R.S., Acevedo C., Arzimanoglou A., Bogacz A., Cross J.H., Elger C.E., Engel J.J., Forsgren L., French J.A., Glynn M. (2014). ILAE official report: A practical clinical definition of epilepsy. Epilepsia.

[2] Kwan P., Arzimanoglou A., Berg A.T., Brodie M.J., Allen Hauser W., Mathern G., Moshé S.L., Perucca E., Wiebe S., French J. (2010). Definition of drug resistant epilepsy: Consensus proposal by the ad hoc Task Force of the ILAE Commission on Therapeutic Strategies. Epilepsia.

[3] Löscher W., Potschka H., Sisodiya S.M., Vezzani A. (2020). Drug Resistance in Epilepsy: Clinical Impact, Potential Mechanisms, and New Innovative Treatment Options. Pharmacol. Rev..

[4] Liu R., Wang J., Liang S., Zhang G., Yang X. (2020). Role of NKCC1 and KCC2 in Epilepsy: From Expression to Function. Front. Neurol..

[5] McMoneagle E., Zhou J., Zhang S., Huang W., Josiah S.S., Ding K., Wang Y., Zhang J. (2024). Neuronal K^+^-Cl^−^ cotransporter KCC2 as a promising drug target for epilepsy treatment. Acta Pharmacol. Sin..

[6] Nguyen T.D., Ishibashi M., Sinha A.S., Watanabe M., Kato D., Horiuchi H., Wake H., Fukuda A. (2023). Astrocytic NKCC1 inhibits seizures by buffering Cl^−^ and antagonizing neuronal NKCC1 at GABAergic synapses. Epilepsia.

[7] Santos L.E.C., Almeida A.G., Silva S.C.B., Rodrigues A.M., Cecílio S.G., Scorza C.A., Finsterer J., Moret M., Scorza F.A. (2023). The amygdala lesioning due to status epilepticus-Changes in mechanisms controlling chloride homeostasis. Clinics.

[8] Palma E., Amici M., Sobrero F., Spinelli G., Di Angelantonio S., Ragozzino D., Mascia A., Scoppetta C., Esposito V., Miledi R. (2006). Anomalous levels of Cl- transporters in the hippocampal subiculum from temporal lobe epilepsy patients make GABA excitatory. Proc. Natl. Acad. Sci. USA.

[9] Sen A., Martinian L., Nikolic M., Walker M.C., Thom M., Sisodiya S.M. (2007). Increased NKCC1 expression in refractory human epilepsy. Epilepsy Res..

[10] Savardi A., Borgogno M., De Vivo M., Cancedda L. (2021). Pharmacological tools to target NKCC1 in brain disorders. Trends Pharmacol. Sci..

[11] Löscher W., Kaila K. (2022). CNS pharmacology of NKCC1 inhibitors. Neuropharmacology.

[12] Töllner K., Brandt C., Römermann K., Löscher W. (2015). The organic anion transport inhibitor probenecid increases brain concentrations of the NKCC1 inhibitor bumetanide. Eur. J. Pharmacol..

[13] Carman A.J., Mills J.H., Krenz A., Kim D.G., Bynoe M.S. (2011). Adenosine receptor signaling modulates permeability of the blood-brain barrier. J. Neurosci..

[14] Kim D.G., Bynoe M.S. (2016). A2A adenosine receptor modulates drug efflux transporter P-glycoprotein at the blood-brain barrier. J. Clin. Investig..

[15] Zavala-Tecuapetla C., Orozco-Suarez S., Manjarrez J., Cuellar-Herrera M., Vega-Garcia A., Buzoianu-Anguiano V. (2020). Activation of adenosine receptors modulates the efflux transporters in brain capillaries and restores the anticonvulsant effect of carbamazepine in carbamazepine resistant rats developed by window-pentylenetetrazole kindling. Brain Res..

[16] Fernandez M., Nigro M., Travagli A., Pasquini S., Vincenzi F., Varani K., Borea P.A., Merighi S., Gessi S. (2023). Strategies for Drug Delivery into the Brain: A Review on Adenosine Receptors Modulation for Central Nervous System Diseases Therapy. Pharmaceutics.

[17] Potschka H., Fedrowitz M., Löscher W. (2003). Multidrug resistance protein MRP2 contributes to blood-brain barrier function and restricts antiepileptic drug activity. J. Pharmacol. Exp. Ther..

[18] Yao D., Liu L., Jin S., Li J., Liu X.D. (2012). Overexpression of multidrug resistance-associated protein 2 in the brain of pentylenetetrazole-kindled rats. Neuroscience.

[19] García-Rodríguez C., Mujica P., Illanes-González J., López A., Vargas C., Sáez J.C., González-Jamett A., Ardiles Á.O. (2023). Probenecid, an Old Drug with Potential New Uses for Central Nervous System Disorders and Neuroinflammation. Biomedicines.

[20] González-Guevara E., Lara-González E., Rendon-Ochoa E., Franco-Pérez J., Hernández-Cerón M., Laville A., Pérez-Severiano F., Martínez-de Los Santos C., Custodio V., Bargas J. (2024). Inhibition of the NMDA Currents by Probenecid in Amygdaloid Kindling Epilepsy Model. Mol. Neurobiol..

[21] Virtanen M.A., Uvarov P., Hübner C.A., Kaila K. (2020). NKCC1, an Elusive Molecular Target in Brain Development: Making Sense of the Existing Data. Cells.

[22] Puskarjov M., Kahle K.T., Ruusuvuori E., Kaila K. (2014). Pharmacotherapeutic targeting of cation-chloride cotransporters in neonatal seizures. Epilepsia.

[23] Römermann K., Fedrowitz M., Hampel P., Kaczmarek E., Töllner K., Erker T., Sweet D.H., Löscher W. (2017). Multiple blood-brain barrier transport mechanisms limit bumetanide accumulation, and therapeutic potential, in the mammalian brain. Neuropharmacology.

[24] Kharod S.C., Kang S.K., Kadam S.D. (2019). Off-Label Use of Bumetanide for Brain Disorders: An Overview. Front. Neurosci..

[25] Töllner K., Brandt C., Töpfer M., Brunhofer G., Erker T., Gabriel M., Feit P.W., Lindfors J., Kaila K., Löscher W. (2014). A novel prodrug-based strategy to increase effects of bumetanide in epilepsy. Ann. Neurol..

[26] Töpfer M., Töllner K., Brandt C., Twele F., Bröer S., Löscher W. (2014). Consequences of inhibition of bumetanide metabolism in rodents on brain penetration and effects of bumetanide in chronic models of epilepsy. Eur. J. Neurosci..

[27] Reid G., Wielinga P., Zelcer N., De Haas M., Van Deemter L., Wijnholds J., Balzarini J., Borst P. (2003). Characterization of the transport of nucleoside analog drugs by the human multidrug resistance proteins MRP4 and MRP5. Mol. Pharmacol..

[28] VanWert A.L., Gionfriddo M.R., Sweet D.H. (2010). Organic anion transporters: Discovery, pharmacology, regulation and roles in pathophysiology. Biopharm. Drug Dispos..

[29] Donovan M.D., O’Brien F.E., Boylan G.B., Cryan J.F., Griffin B.T. (2015). The effect of organic anion transporter 3 inhibitor probenecid on bumetanide levels in the brain: An integrated in vivo microdialysis study in the rat. J. Pharm. Pharmacol..

[30] Zhang G., Franklin P.H., Murray T.F. (1990). Anticonvulsant effect of N-ethylcarboxamidoadenosine against kainic acid-induced behavioral seizures in the rat prepiriform cortex. Neurosci. Lett..

[31] Adami M., Bertorelli R., Ferri N., Foddi M.C., Ongini E. (1995). Effects of repeated administration of selective adenosine A1 and A2A receptor agonists on pentylenetetrazole-induced convulsions in the rat. Eur. J. Pharmacol..

[32] Cai X., Yang L., Zhou J., Zhu D., Guo Q., Chen Z., Chen S., Zhou L. (2013). Anomalous expression of chloride transporters in the sclerosed hippocampus of mesial temporal lobe epilepsy patients. Neural Regen. Res..

[33] Gharaylou Z., Tafakhori A., Agah E., Aghamollaii V., Kebriaeezadeh A., Hadjighassem M. (2019). A Preliminary Study Evaluating the Safety and Efficacy of Bumetanide, an NKCC1 Inhibitor, in Patients with Drug-Resistant Epilepsy. CNS Drugs.

[34] Li X., Zhou J., Chen Z., Chen S., Zhu F., Zhou L. (2008). Long-term expressional changes of Na^+^-K^+^-Cl^−^ co-transporter 1 (NKCC1) and K^+^-Cl^−^ co-transporter 2 (KCC2) in CA1 region of hippocampus following lithium-pilocarpine induced status epilepticus (PISE). Brain Res..

[35] Sivakumaran S., Maguire J. (2016). Bumetanide reduces seizure progression and the development of pharmacoresistant status epilepticus. Epilepsia.

[36] Kourdougli N., Pellegrino C., Renko J.M., Khirug S., Chazal G., Kukko-Lukjanov T.K., Lauri S.E., Gaiarsa J.L., Zhou L., Peret A. (2017). Depolarizing γ-aminobutyric acid contributes to glutamatergic network rewiring in epilepsy. Ann. Neurol..

[37] Bonet-Fernández J.M., Tranque P., Aroca-Aguilar J.D., Muñoz L.J., López D.E., Escribano J., de Cabo C. (2023). Seizures regulate the cation-Cl^−^ cotransporter NKCC1 in a hamster model of epilepsy: Implications for GABA neurotransmission. Front. Neurol..

[38] Othman M.Z., Mohd Nasir M.H., Wan Ahmad W.A.N., Abdullah J.M., Che Has A.T. (2025). Differential regulation of KCC2 and NKCC1 expression by zolpidem in CA1 and CA3 hippocampal subregions of the lithium-pilocarpine status epilepticus rat model. Exp. Anim..

[39] Muñoz A., Méndez P., DeFelipe J., Alvarez-Leefmans F.J. (2007). Cation-chloride cotransporters and GABA-ergic innervation in the human epileptic hippocampus. Epilepsia.

[40] Ding Y., Wang S., Jiang Y., Yang Y., Zhang M., Guo Y., Wang S., Ding M.P. (2013). Fructose-1,6-diphosphate protects against epileptogenesis by modifying cation-chloride co-transporters in a model of amygdaloid-kindling temporal epilepticus. Brain Res..

[41] Eftekhari S., Mehrabi S., Soleimani M., Hassanzadeh G., Shahrokhi A., Mostafavi H., Hayat P., Barati M., Mehdizadeh H., Rahmanzadeh R. (2014). BDNF modifies hippocampal KCC2 and NKCC1 expression in a temporal lobe epilepsy model. Acta Neurobiol. Exp..

[42] González M.I. (2016). Regulation of the cell surface expression of chloride transporters during epileptogenesis. Neurosci. Lett..

[43] Chen L., Wan L., Wu Z., Ren W., Huang Y., Qian B., Wang Y. (2017). KCC2 downregulation facilitates epileptic seizures. Sci. Rep..

[44] Hui K.K., Chater T.E., Goda Y., Tanaka M. (2022). How Staying Negative Is Good for the (Adult) Brain: Maintaining Chloride Homeostasis and the GABA-Shift in Neurological Disorders. Front. Mol. Neurosci..

[45] Sica D.A. (2004). Diuretic-related side effects: Development and treatment. J. Clin. Hypertens..

[46] Ding D., Liu H., Qi W., Jiang H., Li Y., Wu X., Sun H., Gross K., Salvi R. (2016). Ototoxic effects and mechanisms of loop diuretics. J. Otol..

[47] Cheung D.L., Cooke M.J., Goulton C.S., Chaichim C., Cheung L.F., Khoshaba A., Nabekura J., Moorhouse A.J. (2022). Global transgenic upregulation of KCC2 confers enhanced diazepam efficacy in treating sustained seizures. Epilepsia.

[48] Jarvis R., Josephine Ng S.F., Nathanson A.J., Cardarelli R.A., Abiraman K., Wade F., Evans-Strong A., Fernandez-Campa M.P., Deeb T.Z., Smalley J.L. (2023). Direct activation of KCC2 arrests benzodiazepine refractory status epilepticus and limits the subsequent neuronal injury in mice. Cell Rep. Med..

[49] Shi J., Xin H., Shao Y., Dai S., Tan N., Li Z., Fei F., Wu D., Wang Y., Ping Y. (2023). CRISPR-Based KCC2 Upregulation Attenuates Drug-Resistant Seizure in Mouse Models of Epilepsy. Ann. Neurol..

[50] Zavala-Tecuapetla C., Manjarrez-Marmolejo J., Ramírez-Jarquín J.O., Rivera-Cerecedo C.V. (2022). Eslicarbazepine, but Not Lamotrigine or Ranolazine, Shows Anticonvulsant Efficacy in Carbamazepine-Resistant Rats Developed by Window-Pentylenetetrazole Kindling. Brain Sci..

[51] Hubrecht R.C., Carter E. (2019). The 3Rs and Humane Experimental Technique: Implementing Change. Animals.

[52] Davoudi M., Shojaei A., Palizvan M.R., Javan M., Mirnajafi-Zadeh J. (2013). Comparison between standard protocol and a novel window protocol for induction of pentylenetetrazol kindled seizures in the rat. Epilepsy Res..

[53] Racine R.J. (1972). Modification of seizure activity by electrical stimulation. II. Motor seizure. Electroencephalogr. Clin. Neurophysiol..

[54] Corda M.G., Giorgi O., Longoni B., Orlandi M., Biggio G. (1990). Decrease in the function of the gamma-aminobutyric acid-coupled chloride channel produced by the repeated administration of pentylenetetrazol to rats. J. Neurochem..

[55] Srivastava A.K., Alex A.B., Wilcox K.S., White H.S. (2013). Rapid loss of efficacy to the antiseizure drugs lamotrigine and carbamazepine: A novel experimental model of pharmacoresistant epilepsy. Epilepsia.

[56] Brandt C., Nozadze M., Heuchert N., Rattka M., Löscher W. (2010). Disease-modifying effects of phenobarbital and the NKCC1 inhibitor bumetanide in the pilocarpine model of temporal lobe epilepsy. J. Neurosci..

